# 

**Published:** 1888-01

**Authors:** 


### INDEX

TO THE

### HOMŒOPATHIC PHYSICIAN.

### VOLUME VIII.

PAGE

Abrotanum, with Clinical Cases. J. T. Kent, M. D., . . . . . . . . . . . . . 58
A[illegible]ic Acid, . . . . . . . . . . . . . . 142
[illegible]nite. A. B. Eadie, M. D., . . . . 259
The Abuse of (Note), . . . . . . . 276
Post-Partum and other Hemorrhages. Hamilton Evans, M. D., 298
[illegible]n., . . . 92, 93, 109, 130, 142, 172, 298, 357, 383, 405, 425, 453, 519, 538, 545
A[illegible]ea racemosa, . . . . . . . . . . 132, 407
Actea Spicata, . . . . . . . . . . . . 80
Adams, Edward, M. D. China Remedies for Post-Partum Hemorrhage, . 121
Cina, . . . . . . . . . . . . . . . 256
Hamamelis. Post-Partum and other Hemorrhages, . . . . . . . . . 298
Helleborus, . . . . . . . . . . . . . 421
Helonias Dioica. Post-Partum and other Hemorrhages, . . . . . . 297
Hyoscyamus. Post-Partum and other Hemorrhages, . . . . . . 297
Pulsatilla Remedies for Post-Partum Hemorrhages, . . . . . . . 202
Address on Hospital and Dispensary Clinics and the Art of Prescribing. By Prosper Bender, M. D. Review of, . . . . . . . . . . . . . . . . . 386
Address before the International Hahnemannian Association at its Ninth Annual Meeting. Dr. Wm. P. Wesselhœft, . . . . . . . . . . . . . . . 387
Adhesions following Pelvic Peritonitis. J. M. Dutton, M. D., . . . . . . 383
Ægidi, . . . . . . . . . . . . . . . 205
Æsculus Hippocastanum: Therapeutics of the Throat. John V. Allen, M. D., . . . . . . . . . . . . . . . . 64
Æsculus Hippocastanum, . . . 64, 81, 142, 365
Æthusa, . . . . . . . . . . . . 83, 282, 589
Agaricus Emeticus, . . . . . . . . . . 86
Agaricus Muscarius. Clinical Notes on Characteristics of. C. Carleton Smith, M. D., and E. J. Lee, M. D., . . . . 144
Agaricus musc., . . . . . 118, 125, 170, 377
Agnus Castus. Clinical Notes on Characteristics. E. J. Lee, M. D., and C. Carleton Smith, M. D., . . . . . . . 150
Agnus castus, . . . . . . . . . . . 142, 589
Ailanthus. Clinical Notes on Characteristics. C. C. Smith, M. D., and E. J. Lee, M. D., . . . . . . . . . . . . 218
Ailanthus Glandulosa. John V. Allen, M. D., . . . . . . . . . . . . . . . . . 67
Aletris Formosa. Clinical Notes on Characteristics. C. Carleton Smith, M. D., and E. J. Lee, M. D., . . . . 221
Allen, Professor T. F., M. D. Lecture upon the *Organon*, . . . . . . . . . 1
Allen, John V., M. D. Ailanthus Glandulosa, . . . . . . . . . . . . . . . 67
Allen, John V., M. D. Therapeutics of the Throat; Æsculus Hip., . . . . 64
Allen, John V., M. D. Carcinoma, . 656
Allium Cepa. Clinical Notes on Characteristics. C. Carleton Smith, M. D., and Edmund J. Lee, M. D., . . . 223
Allium Sativa. Clinical Notes on Characteristics. C. Carleton Smith, M. D., and Edmund J. Lee, M. D., . . 225
Aloe Socotrina, an Anti-psoric Remedy. W. P. Wesselhœft, M. D., . . . . . . 576
Aloes, . . . . . . . 42, 73, 110, 282, 580, 589
Alumen, . . . . . . . . . . . . . . 44, 108
Alumina, . . . . 365, 439, 530, 551, 569, 589
Ambra Grisea. Horace Still, M. D., . 126
Ambra, . . . . . . . . . . 70, 125, 129, 589
American Institute of Homœopathy, 275, 450
American Institute of Homœopathy; Transactions of; Review of, . . 48, 667
Ammon. brom., . . . . . . . . . . . 67
Ammonium carb., . . . 40, 69, 94, 125, 142
Ammon. mur., . . . . . . 93, 127, 365, 589
Anacardium, . . . . 126, 142, 365, 589, 597
Analytic Study. Edward Fornias, M. D., . . . . . . . . . . . . . . . . 485
Anchylosis, Remediable. Geo. H. Clark, M. D., . . . . . . . . . . . . . 418
Andrews, Dr. H. W. Clinical Notes, . 185
Annual of the Universal Medical Sciences. Dr. Charles E. Sajous. Review of, . . . . . . . . . . . . . . . 619
Another, . . . . . . . . . . . . . . . 158
Another Medical School, . . . . . . . . 450
Answer to Dr. Jamison's Case for Counsel. F. H. Lutze, M. D., . . . . 131
Anthracinum, . . . . . . . . . . . . 573
Antimonium crudum, . . . . . . . . 365
Antimon. tart., . . . . . . . . . . 130, 453
"Aphorisms," Bœnninghausen's. Translated by Wm. P. Wesselhœft, M. D., 32
Apis Mellifica. Post-Partum and other Hemorrhages. J. D. Tyrrell, M. D., . 296

PAGE
Apis. W. J. Hunter Emory, M. D., . . 420
Apis, . . . . 4, 76, 117, 131, 132, 185, 197, 265, 270, 281, 352, 372, 383, 446, 618
Apocynum Cannabinum. Post-Partum and other Hemorrhages. W. J. Hunter Emory, M. D., . . . . . . . 296
Apocynum, . . . . . . . . . . . . . . . 305
Aqua Sanicula, Some Clinieal Notes Upon. G. W. Sherbino, M. D., . . . 439
Argentum, . . . . . . . . . . . . 153, 185
Argent. nit., . . . 68, 77, 243, 326, 466, 589, 598, 654
Arnica Montana. Post-Partum and other Hemorrhages. W. J. Hunter Emory, M. D., . . . . . . . . . . . 296
Arnica, . . . . 14, 67, 109, 142, 549, 589, 616
Arsenicum alb., . . . 2, 15, 19, 103, 109, 127, 142, 183, 185, 263, 270, 311, 313, 327, 407, 439, 468, 496, 571, 589, 665
Ars. iodid., . . . . . . . . . . . . . . 138
Artesnisia-vulg., . . . . . . . . . . . 598
Arum-tri., . . . . . . . . . . . 15, 290, 328
Asafœtida, . . . . . . . . . . . . 589, 598
Asphyxia Neonatorum, . . . . . . . . 130
Asterias Rubens. Geo. H. Clark, M. D., . . . . . . . . . . . . . . . . . 20
Asterias rubens, . . . . . . . . . . . 18
Atlantic City, . . . . . . . . . . . . 449
Atropinum, . . . . . . . . . . . 67, 545
Aurum, . . . . . . . . . . . . . . 125, 589

Baer, Dr. O. P. Notice of Death of, . 532
Baker, W. H., M. D. Hydrastic Canadensis, . . . . . . . . . . . . . . . . 243
Baker, W. H., M. D. The Rochester Hahnemannian Society, . . 242, 360, 496, 538
Ballard, Dr. E. A. Notice of, . . . . . 450
Baptisia, . . . . . . . 15, 180, 181, 196, 242
Baruch, Dr. A Note on Theridion. . . 331
Baryta carb., . . . . . . . 62, 170, 553, 589
Bastinado for Asphyxia. Ida F. Norris, M. D., . . . . . . . . . . . . . . 89
Bayard, Edward, M. D. A Clinical Case, with Comments, . . . . . . . 616
Baylies, B. L. B., M. D. Clinical Confirmation of High Potencies, . . . . 40
Baylies, B. L. B., M. D., Sepia. Therapeutics of the Throat, . . . . . . . . . . 231
Belladonna. John Hall, Sr., M. D., . 253
Belladonna. Remedies for Post-Partum Hemorrhage. Hamilton Evans, M. D., . . . . . . . . . . . . . . 119
Belladonna, . . . 19, 41, 93, 101, 110, 130, 137, 141, 142, 149, 183, 196, 210, 265, 270, 272, 281, 316, 328, 344, 372, 373, 407, 455, 497, 528, 545, 660
Bell on Diarrhœa. Note of, . . . . . 275
Bender, Prosper, M. D. Address upon Hospital and Dispensary Clinics and the Art of Prescribing, . . . . . . . 386
Berberis Case, A. B. Simmons, M. D., 650
Berberis, . . . . . . . 41, 69, 361, 569, 650
Berridge, E. W., M. D. Clinical Cases and Provings, . . . . . . . . . . . 547
Fish Brine Odor, . . . . . . . . . 185
A Case of Suppressed Gonorrhœa, 315
A Gynæcological Case, . . . . . . 425
Matteism, the latest Craze, . . . 560
Bœnninghausen. Tabes Dorsalis and Aluminum Metallicum. . . . . . . 526
Bœnninghausen's Aphorisms. Translation of. Wm. P. Wesselhœft, M. D., . . . . . . . . . . . . . . . . 32
Book Notices and Reviews, . . 48, 95, 157, 211, 275, 331, 386, 448, 506, 556, 619, 667

PAGE
Borax, . . . . . . . . . . . . . . . . 176
Bovista, . . . . . . . . . . . . . . . 545
Bromium, . . . . . . . . . . . . . . 138
Brownell, Dr. Report of a Case of Diphtheria, . . . . . . . . . . . . . 541
Bryonia Alba, Post-Partum and other Hemorrhages. W. J. Hunter Emory, M. D., . . . . . . . . . . . . . . . 297
Bryonia, . . . 5, 16, 91, 101, 109, 110, 140, 155, 173, 185, 206, 354, 356, 366, 372, 455, 528, 538, 554, 589, 639
Bufo, . . . . . . . . . . . . . . . . 545
Butler, Clarence Willard, M. D. Clinical Notes, . . . . . . . . . . . . . 93
Butler, Clarence Willard, Sec. Proceedings of the New York Hom. Union, 299, 486
Butler, Clarence W., M. D. Locomotor Ataxy—A Clinical Case, . . . . . . 651

Cactus, . . . . . . . . . . . . . . . . 589
Cajeput, . . . . . . . . . . . . . . . 593
Caladium, . . . . . . . . . . . . . . 153
Calcarea. Remedies for Post-Partum Hemorrhage. J. D. Tyrrell, M. D., . 120
Calcarea carb., . . . 101, 149, 153, 173, 185, 268, 282, 446, 524, 548, 553, 589, 598, 649
Calcarea fluorica, . . . . . . . . . . . 169
Calcarea phosphorica, . . . 59, 69, 169, 171
Calcarea sulph., . . . . . . . . . . . 171
Calendula, . . . . . . . . . . . . 109, 142
California Homœopath, The. Notice of, . . . . . . . . . . . . . . . . . 48
Cameron, Elizabeth W. M., M. D. Clinical Notes, . . . . . . . . . . . . . 370
Campbell, Alice B., M. D. A Peculiar Case, . . . . . . . . . . . . . . . 665
Camphora, . . . . . . . . . . . . . . 425
Cancer, A Diagnostic Sign of. A. Mc.-Neil, M. D., . . . . . . . . . . . . . 130
Cannabis-ind., . . . . . . . . . 149, 185, 545
Cantharis, . . . . . . . . 142, 205, 572, 573
Capsicum, . . . . . . . . . . . . . . 185, 247
Carbo Animalis. Therapeutics of the Throat. E. Cranch, M. D., . . . . . 226
Carbo animalis, . . . . . . . . . . 143, 407
Carbo Vegetabilis. Therapeutics of the Throat. E. Cranch, M. D., . . . . . 228
Carbo veg., . . . 4, 16, 127, 325, 552, 574, 641
Carbn. s., . . . . . . . . . . . . . . . 67
Carcinoma. John V. Allen, M. D., . . 656
Carlsbad, . . . . . . . . . . . . . . . 594
Case for Counsel, A. M. R. Jamison, M. D., . . . . . . . . . . . . . . . . 87
Case, A Peculiar. Alice B. Campbell, M. D., . . . . . . . . . . . . . . . 665
Cases from Practice. B. Simmons, M. D., . . . . . . . . . . . . . . . . . 648
Caulophyllum, Post-Partum and other Hemorrhages. Hamilton Evans, M. D., . . . . . . . . . . . . . . . . . . 299
Cauloph, . . . . . . . . . . . 243, 361
Causticum, Analytic Study of Edward Fornias, M. D., . . . . . . . . 485
Causticum, . . . 76, 407, 428, 528, 550, 575, 589, 598
Central New York Homœopathic Medical Society, Proceedings of, . . . . 100
Cepa, . . . . . . . . . . . . . . . . . 108
Cerebral Congestion. William Hawley, M. D., . . . . . . . . . . . . . 155
Chamomilla in Puerperal and other Convulsions. L. Hamilton Evans, M. D., . . . . . . . . . . . . . . . 258
Chamomilla, Post-Partum and other Hemorrhages. J. D. Tyrrell, M. D., 295

PAGE
Chamomilla, . . . 97, 183, 208, 453, 456, 589
Characteristics, Clinical Notes on. See "Clinical Notes on Characteristics."
Characteristics of the Ten Tissue Remdies. E. J. L., . . . . . . . . . . . . 169
Characteristics as Taught by Hahnemann. Edmund J. Lee, M. D., . . 451
Chase, C. E., M. D. Rhus Toxicodendron. Therapeutics of the Throat, . 193
Chelidonium. C. Carleton Smith, M. D., . . . . . . . . . . . . . . . 185
Chelidonium, . . . . . . . . . . 185, 366, 589
China. Remedies for Post-Partum Hemorrhage. Edward Adams, M. D., 121
China, . . . 4, 16, 104, 111, 121, 153, 170, 204, 205, 283, 286, 455, 456, 505, 553, 589
Chininum Sulph., A Case of Fatal Poisoning by. S. L., . . . . . . . . 179
Chin. sulph., . . . . . . . . . . . . . 69
Choice of a Doctor, The, . . . . . . . 96
Chronic Diseases. E. J. L., . . . . . . 335
Cicuta Virosa. W. J. Hunter Emory, M. D., . . . . . . . . . . . . . . . . 254
Cicuta Virosa, A Verification of. Alfred Heath, Esq., . . . . . . . . . . . . . 186
Cicuta, . . . . . . . . . . . 125, 149, 186, 598
Cimic, . . . . . . . . . . . . . . . . . 149
Cina. Edward Adams, M. D., . . . . 256
Cina, . . . . . . . . . . . . . 32, 149, 644
Cinchona, . . . . . . . . . . . . . . 205
Cinnamonium. Remedies for Post-Partum Hemorrhage. John Hall, Sr., M. D., . . . . . . . . . . . . . . . . 203
Circulation of the Blood, Forces Engaged in. C. W. Spalding, M. D. . . 542
Cistus. Therapeutics of the Throat. E. J. Lee, M. D., . . . . . . . . . . . . . 8
Cistus can., . . . . . . . . . . 4, 68, 69
Clark, Geo. H., M. D. Remediable Anchylosis, . . . . . . . . . . . . . 418
Clark, Geo. H., M. D. Asterias Rubens, 20
Clark, Geo. H., M. D. In Memoriam Ad. Fellger, M. D., . . . . . . . . . 501
Clark, Geo. H., M. D. Therapeutics of Throat. Ignatia, . . . . . . . . . 9
Clark, Geo. H., M. D. Some Symptoms of Sycosis, . . . . . . . . . . . . . 398
Clark, Geo. H., M. D. Typhoid Fever, 635
Class-Room Talks, from Lectures of Prof. J. T. Kent. S. L. G. L.. . . . 34, 97, 239, 324, 343, 474
Clinical Cases. F. E. Stoaks, M. D., . 438
Clinical Cases. Stuart Close, M. D., 91, 329
Clinical Case, with Comments, A. Edward Bayard, M. D., . . . . . . . . 616
Clinical Cases and Provings. E. W. Berridge, M. D., . . . . . . . . . . . 547
Clinical Cases, Some. Geo. W. Sherbino, M. D., . . . . . . . . . . . . . 179
Clinical Case with Appendices. J. N. Lowe, M. D., . . . . . . . . . . . 137
Clinical Notes. Elizabeth W. M. Cameron, M. D., . . . . . . . . . . . . . 370
Clinical Note, A, . . . . . . . . . . . 328
Clinical Notes. H. W. Andrews, M. D., 185
Clinical Notes Clarence Willard Butler, M. D., . . . . . . . . . . . . . . 93
Clinical Notes on Characteristics. C. Carleton Smith, M. D., and Edmund J. Lee, M. D., . . . . . . . . 39, 80, 144, 218
Clinical Report of Three Cases Treated by R. B. Johnstone, M. D., . . . . . 660
Clinical Verifications. H. C. Morrow, M. D., . . . . . . . . . . . . . . . . 305
Clinical Work, . . . . . . . . . . . . 334

PAGE
Close, Stuart, M. D. Clinical Cases, 91, 329
Close, Stuart, M. D. Tracheotomy—A Plea for Natural Death, . . . . . . . 340
Cobalt, . . . . . . . . . . . . . . . . . 154
Colchicum, . . . . . . . . 16, 69, 70, 209, 454
Colocynth, . . . . . . . . . . . 112, 469, 643
Complete Hand-Book of Treatment, by William Aitken, M. D., Notice of. . . 48
Cocaine, A Peculiar Symptom of. Wm. Jefferson Guernsey, M. D., . . . . . 596
Cocculus indicus, . . . . . . . . . . . 41
Coccus cacti, . . . . . . . . . . . . . 316
Codein, . . . . . . . . . . . . . . . . 149
Coffea cr., . . . . . . . . . . . . . . . 95
Cohen, S. W., M. D. Gynæcology, . . 435
Colchicum, . . . . . . . . 16, 69, 70, 209, 454
Colocynth, . . . . . . . . . 112, 183, 439, 469
Comparisons of Aconite with Apis and Gelsemium in Fevers, . . . . . . . 383
Conium, . . . 125, 154, 328, 548, 570, 573, 589, 595, 652
Constipation, and Some of the Most Prominently Indicated Remedies in its Treatment. Alex. C. Hermance, M. D., . . . . . . . . . . . . . . . 364
Constipation, Prolonged. Dr. W. S. Gee, . . . . . . . . . . . . . . . . 43
Contagious Diseases caused by Acute Miasmata. T. Dwight Stow, M. D., . 49
Contradictory Symptoms, . . . . . . . . 327
Contributions to the Study of the Heart and Lungs, by James R. Leaming, M. D., Notice of, . . . . . . . . . . 157
Convulsions, Puerperal and other, 253, 254, 255, 256, 257, 258
Corallum rub., . . . . . . . . . . . . 125
Correction, A. S. L., . . . . . . . . . . 384
Corrections, see "Errata."
Correspondence. Robt. Farley, M. D., 447
Counsel, A Case for. M. R. Jamison, M. D., . . . . . . . . . . . . . . . . . 87
Cranch, Edward, M. D. Carbo Animalis, . . . . . . . . . . . . . . . . . 226
Carbo Vegetabilis, . . . . . . . . 228
Cranch, Edward, M. D. Selections of Characteristic Symptoms, Hahnemann's *Organon*. Paragraph 153, . . 404
Cranch, Edward, M. D. Therapeutics of the Throat, . . . . . . . . . . . . 226
Criticism of Dr. Holmes, A. J. T. Kent, M. D., . . . . . . . . . . . . . . . . 639
Crocus Sativus. Remedies for Post-Partum Hemorrhage. W. J. Hunter Emory, M. D., . . . . . . . . . . . 122
Crocus sativus, . . . . . . . . . 41, 143, 598
Crotalus, . . . . . . . . . . . . . . . 102
Croton Tiglium. J. H. Hamer, M. D., 320
Crot-tig., . . . . . . . . . . . . . . . 235
Croup and its Management. By Thomas Nichol, M. D. Review of, . . . . 386
Cup-m., . . . . . . . . . . . . . . . . 589

Diabetes, Physiology and Pathology of. Notice of, . . . . . . . . . . . . . . 386
Diagnostic Remarks, Some. J. M. Miller, M. D., . . . . . . . . . . . . . . 615
Dietetics. Its Bearing on Special and General Tissue Building. W. J. Thayer, D. D. S., M. D., . . . . . . 346
Dietetic Case, A. A. McNeil, M. D., . . 345
Dietetic Case, Notes on a. S. L., . . . 479
Digitalis, . . . . . . . . . . 76, 154, 207, 616
Diphtheria with Convulsions Cured, A Case of. William H. Krause, M. D., . . . . . . . . . . . . . . . . . 271

PAGE
Diphtheria, Report of a Case of. Dr. Brownell, . . . . . . . . . . . . . . 541
Direction of Symptoms, The, . . . . . 438
Discussion from Transactions of I. H. A., 1888, . . . . . . . . . . . . 586
Disease, A Terrible, . . . . . . . . . 59
Domestic Cook Book. By Mrs. J. H. Pulte. Notice of, . . . . . . . . . . 448
Dose, The. P. P. Wells, M. D., . . . . 623
Drug, How to find the. N. W. Vandenburg, M. D., . . . . . . . . . . . . 632
Dulcamara, Lecture on. Dr. A. McNeil, . . . . . . . . . . . . . . . . 60
Dulcamara, . . . . . . . . . . . . 17, 546
Dutton, J. M., M. D. Adhesions following Pelvic Peritonitis, . . . . . 382
Dutton, J. M., M. D. Sulphur in Melancholia, . . . . . . . . . . . . . . 380
Dysentery, A Case of. Rev. J. K. Mendenhall, . . . . . . . . . . . . . 90
Dyspnœa, Fresh Air, . . . . . . . . . 4

Eadie, A. B., M. D. Aconite, . . . . . 259
Eadie, A. B. Ferrum, Remedies for Post-Partum Hemorrhage, . . . . . 123
Eadie, A. B., M. D., Nux Vomica, . . 423
Eadie, A. B., M D., Phosphorus. Remedies for Post-Partum Hemorrhage, 201
Eadie, A. B., M. D. Platina. Post-Partum and other Hemorrhages, . . 293
Eadie, A. B., M. D. Stramonium. Post-Partum and other Hemorrhages, . . 294
Eadie, A. B., M. D. Sulphuric Acid. Post-Partum and other Hemorrhages, 294
Editorial Notes. E. J. Lee, M. D., . . 621
Elliott, J. B., M. D. A Case of Epithelioma, . . . . . . . . . . . . . . . 533
Emory, W. J. Hunter, M. D. Apis, . . 420
Emory, W. J. Hunter, M. D. Apocynum Cannabinum. Post-Partum and other Hemorrhages, . . . . . . . . 296
Emory, W. J. Hunter, M. D. Arnica Montana. Post-Partum and other Hemorrhages, . . . . . . . . . . . 296
Emory, W. J. Hunter. Bryonia Alba. Post-Partum and other Hemorrhages, 297
Emory, W. J. Hunter, M. D. Cicuta Virosa, . . . . . . . . . . . . . . . 254
Emory, W. J. Hunter, M. D. Crocus Sativus. Remedies for Post-Partum Hemorrhage, . . . . . . . . . . . 122
Emory, W. J. Hunter, M. D. Sabina. Remedies for Post-Partum Hemorrhage, . . . . . . . . . . . . . . . 204
Enuresis, A Case of Nocturnal, . . . 273
Epithelioma, A Case of. J. B. Elliott, M. D., . . . . . . . . . . . . . . . 533
Erigeria. Post-Partum and other Hemorrhages. Hamilton Evans, M. D., 299
Errata, . . . 213, 275, 450, 506, 554, 555, 556, 576, 620
Erysipelas, A Case of. H. P. Holmes, M. D., . . . . . . . . . . . . . . . 270
Eupatorium-per., . . . . . . . . . . . 454
Euphorbium, . . . . . . . . . . . . . 206
Euphrasia, . . . . . . . . . . . . 185, 546
Evans, Hamilton, M. D. Aconite. Post-Partum and other Hemorrhages, 298
Evans, Hamilton, M. D. Belladonna. Remedies for Post-Partum Hemorrhage, . . . . . . . . . . . . . . . 119
Evans, Hamilton, M. D. Caulophyllum. Post-Partum and other Hemorrhages, . . . . . . . . . . . . . . 299
Evans, Hamilton, M. D. Chamomilla in Puerperal and other Convulsions, 258
Evans, Hamilton, M. D. Erigeria. Post-Partum and other Hemorrhages, 299
Evans, Hamilton, M. D. Hyoscyamus, 422
Evans, Hamilton, M. D. Ustilago. Remedies for Post-Partum Hemorrhage, . . . . . . . . . . . . . . . 200
Examination of the Patient for a Homœopathic Prescription, The. P. P. Wells, M. D., . . . . . . . . . 509
Excessive Venery, Masturbation, etc. By Joseph W. Howe, M. D. Review of, . . . . . . . . . . . . . . . . 556
Experience, A First. F. M. Gustin, M. D., . . . . . . . . . . . . . . . . 182

Faith Cures, . . . . . . . . . . . . . 276
Farley, Robt., M. D. Correspondence, 447
Farley, Robt., M. D. Rhus Rad. *versus* Rhus Tox., . . . . . . . . . . 313
Farley, Robt., M. D. Taking the Case, 25
Ferrum. Remedies for Post-Partum Hemorrhage. A. B. Eadie, M. D. . . 123
Ferrum, . . . . . . . . . . . 170, 470, 616
Ferrum phos., . . . . . . . . . . . . 172
Fifty Reasons for Being a Homœopath. By Compton Burnett, M. D. Review of, . . . . . . . . . . . . . . . . 331
Fish-Brine Odor. E. W. Berridge, M. D., . . . . . . . . . . . . . . . 185
Fitz, W. H. A., M. D. The *Organon*, Section 153, . . . . . . . . . . . . 599
Fluoric acid, . . . . . . . . . . . 127, 453
Fox's Atlas and Text-Book of Skin Diseases, Notice of, . . . 157, 211, 333, 667
For Sale, . . . . . . . . . . . 89, 212, 506
Fornias, Edward, M. D. Analytic Study—Causticum, . . . . . . . . . 485
Fragments. Dr. Flora Waddell, . . . 444
Frisby, A. J., M. D. The Hospitals of the Women's Homœopathic Association of Pennsylvania, . . . . . . . 37

Gamboge, . . . . . . . . . . . . . . . 363
Garrison, J. B., M. D. Report of a Few Cases Terminating Successfully Under the Administration of the Higher Potencies, . . . . . . . . . . . . . 306
Gee, W. S., M. D. Peculiar Symptoms, . . . . . . . . . . . . . . . . 580
Gee, W. S., M. D. Prolonged Constipation, . . . . . . . . . . . . . . . 43
Gelsemium, . . . . . . . . . . 17, 383, 453
Gelsemium nitridum, . . . . . . . . . 42
Gilbert, Chas, B., M. D. Hahnemann's Use of the Tincture, . . . . . . . . 554
Glands. C. W. Spalding, M. D., . . . 458
Glonoine, . . . . . . . . . . . . . 316, 465
Glover, H. G., M. D. A Few Verifications, . . . . . . . . . . . . . . . . 618
Gonorrhœa, A Case of Suppressed. E. W. Berridge, M. D., . . . . . . . . . 315
Gonorrhœa, Repertory of. Saml. A. Kimball, M. D., . . . . . . . . . . 332
Good Surgical Journal, A, . . . . . . 620
Goullon, Dr. H. Fallacious and True Theories Concerning Certain Skin Diseases, . . . . . . . . . . . . . . 480
Graphites, . . . 42, 58, 143, 185, 210, 438, 496, 570, 575, 590
Gratiola, . . . . . . . . . . . . . . . 67
Guernsey's Bœnninghausen, Notice of, 95
Guernsey, Wm. Jefferson, M. D. A Peculiar Symptom of Cocaine, . . . . 596
Guernsey, Wm. Jefferson, M. D. Proceedings of the Hahnemannian Association of Pennsylvania, . . 17, 124, 592

PAGE
Guernsey, Wm. Jefferson, M. D. Resolution of the Hahnemannian Association of Pennsylvania, . . . . . . 501
Guernsey, Wm. Jefferson, M. D. The Quinine Curse, . . . . . . . . . . . . 71
Guernsey, Wm. Jefferson, M. D. Therapeutics of the Throat. Arum Triphyllum, . . . . . . . . . . . . . 132
Gustin, F. M., M. D. A First Experience, . . . . . . . . . . . . . . . . 182
Gynæcological Case, A. E. W. Berridge, M. D., . . . . . . . . . . . . . 425
Gynæcology, S. W. Cohen, M. D., . . 435
Gynæcology, A Text-Book of. By Prof. A. C. Cowperthwaite, M. D. Review of, . . . . . . . . . . . . . . . . . 620
Gynæcology, Practical Manual of. G. R. Southwick. M. D. Review of, . . 157

Hæmatoxylon, . . . . . . . . . . . . 593
Hahnemann Club of Toronto, The, . 6
Hahnemann Club of Toronto, Proceedings of, . . . . . 74, 116, 198, 251, 291, 420
Hahnemannian Association of Pennsylvania, Proceedings of, . . . 17, 69, 124, 501, 592
Hahnemannian Monthly for Sale, . . 668
Hahnemann's Writings and Rubrick, 156
Hahnemann's *Organon*. A Query. S. Lilienthal, M. D., . . . . . . . . . 385
Hahnemann's Use of the Tincture. Chas. B. Gilbert, M. D., . . . . . 554
Hall, John, Sr., M. D. Belladonna, . 253
Hall, John, Sr., M. D. Cinnamonium. Remedies for Post-Partum Hemorrhage, . . . . . . . . . . . . . . . 203
Hall, John, Sr., M. D. Ipecacuanha. Remedies for Post-Partum Hemorrhage, . . . . . . . . . . . . . . . 117
Hamamelis. Post-Partum and other Hemorrhages. Edward Adams, M. D., . . . . . . . . . . . . . . . . . 298
Hamamelis, . . . . . . . . . . . . . 143
Hamer, J. H., M. D. Croton Tiglium, 320
Hawley, Wm. A., M. D. Cerebral Congestion, . . . . . . . . . . . . . . 155
Hawley, W. A., M. D. The Vital Force, 368
Hay Fever, or Rhinitis Vasco-Motor Periodica and its Radical Cure. By E. Lippincott, M. D. Review of, . . 619
Head Symptoms, Repertory of. C. Neidhard, M. D. Review of, . . . . 331
Heart and Lungs, Contributions to the Study of. James R. Leaming, M. D., 157
Heath, Alfred, Esq. A Verification of Cicuta Virosa, . . . . . . . . . . . 186
Helleborus. Edward Adams, M. D., . 421
Hellebore, . . . . . . . . . . . . 328, 590
Helonias Dioica. Post-Partum and other Hemorrhages. Edward Adams, M.D., . . . . . . . . . . . . . . . . . 297
Helon, . . . . . . . . . . . . . . . . 170
Hemorrhages, Post-Partum and other, 117, 119, 122, 123, 200, 201, 204, 258, 293, 294, 296, 297, 298, 299
Henderson, L., M. D. Medical Journals and Practitioners, . . . . . . . 63
Hepar, . . . . . . . . 68 116, 143, 180, 590
Hermance, Alex. C., M. D. Constipation and Some of the Most Prominently Indicated Remedies in its Treatment, . . . . . . . . . . . . . . 364
High Potencies, Clinical Confirmation of. B. L. B. Baylies, M. D., . . . . 40
Holloway, J. C., M. D. A Case of Remittent Fever, . . . . . . . . . . . 274

PAGE
Holmes, H. P., M. D. A Case of Erysipelas, . . . . . . . . . . . . . . . . 270
Holmes, H. P., M. D. Veratrum in Cholera Morbus, . . . . . . . . 602
A Criticism of. J. T. Kent, M. D., 639
Homœopathic Domestic Indicator. Notice of, . . . . . . . . . . . . . . . 96
Homœopathic Law of Similarity. By Dr. von Grauvogl. Review of, . . . 48
Homœopathic League Tracts, Notice of, . . . . . . . . . . . . . . . . . 96, 332
Homœopathic Materia Medica, its Uses and its Imperfections, The. E. J. L., 557
Homœopathic Med. College of Missouri, . . . . . . . . . . . . . . . . 210
Homœopathic Medical Society of Ohio, Proceedings of, . . . . . . . . . . . 211
Homœopathic Medical Society of Penna., Transactions of. Review of, 96
Homœopathic Medical Society of Penna., . . . . . . . . . . . . . . . 645
Homœopathic Physcians' Visiting List and Repertory. Robt. Faulkner, M - D. Notice of, . . . . . . . . . . . . 158
Homœopathic Therapeutics of Diarrhœa, Dysentery, Cholera, Cholera Morbus, and Cholera Infantum. By James B. Bell, M. D. Review of, . . 556
Homœopathic Treatment of Rheumatism and Kindred Diseases. D. C. Perkins, M. D. Review of, . . . . . 158
Homœopathy *vs.* Intermittent Fever. Samuel Long, M. D., . . . . . . . . . 45
Homœopathy in Venereal Diseases. By Stephen Yeldham. Review of, . . . 506
Homœopathy, Montreal Tracts upon, Notice of, . . . . . . . . . . . . . . 668
Hooker, Frederick, M. D. In Memoriam Mrs. Stephen Seward, . . . . 502
Hooker, Frederick, M. D. Proceedings of the Syracuse Hahnemannian Club, . . . . . . . . . 262, 357, 491, 502, 641
Hooker, Frederick, M. D. Queries as to Rhus and other Poisonings, . . . 384
Hospital Clinics, Address upon. Prosper Bender, M. D., . . . . . . . . . 386
Hospital, A Proposed New Homœopathic, . . . . . . . . . . . . . . 647
Hospitals of the Women's Homœopathic Ass'n of Penna. A. J. Frisby, M. D., . . . . . . . . . . . . . . . . . 38
How They were Converted. Hom. League Tract, No. 16, . . . . . . . . 96
How to Find the Drug. N. W. Vandenburg, M. D., . . . . . . . . . . . 632
How is this? . . . . . . . . . . . . . 555
How Symptoms Change. J. T. Kent, M. D., . . . . . . . . . . . . . . . 596
How to Help Us, . . . . . . . . . . . 646
Hughes, Dr., Rejoinder to. By Prosper Bender, M. .D, Review of, . . . . . 668
Hydrastis Canadensis. W. H. Baker, M. D., . . . . . . . . . . . . . . . . 243
Hydrastis, . . . . . . . . . . . . . . 41
Hydrophob., . . . . . . . . . . . . . 584
Hyoscyamus. Post-Partum and other Hemorrhages. Edward Adams, M. D., . . . . . . . . . . . . . . . . 297
Hyoscyamus. Hamilton Evans, M. D., 422
Hyoscyamus, . . . . . . . . . . 41, 71, 149
Hypericum, . . . . . . . . . . . . 143, 243
Hypochondriasis, A Partial Repertory of. N. W. Vandenburg, M. D., . . . 589

Ignatia. Therapeutics of the Throat. George H. Clark, M. D., . . . . . . 9

PAGE

Ignatia, . . . 41, 101, 149, 366, 453, 456, 550, 590, 642
Indented Tongue, . . . . . . . . . . 446
Indium met., . . . . . . . . . . . . . . 592
In Memoriam Ad. Lippe, M. D. Supplement to Feb. No.
In Memoriam Ad. Fellger, M. D. Geo. H. Clark, M. D., . . . . . . . . . . 501
In Memoriam Mrs. Stephen Seward. Frederick Hooker, M. D., . . . . . . 502
International Hahnemannian Association, Address before. Wm. P. Wesselhœft, M. D., . . . . . . . . . 387
International Hahnemannian Association, The, . . . . . . . . . . . . 79
Meeting at Niagara, . . . 333, 394, 417
International Hahnemannian Association, Report of Proceedings of. Review of, . . . . . . . . . . . . . 448
International Hahnemannian Association, New Members for, . . . . . 591
Inquirer, Answer to, . . . . . . . . . 57
Iodine, . . . . . . . . . . . . . . . . 590
Ipecacuanha. Remedies for Post-Partum Hemorrhage. John Hall, Sr., M. D., . . . . . . . . . . . . . . . . 117
Ipecac., . . . . . . . . 117, 118, 185, 247, 358

Jaborandi, . . . . . . . . . . . . 125, 149
Jamison, M. R., M. D. A Case for Counsel, . . . . . . . . . . . . . . 87
Johnstone, R. B., M. D., Clinical Report of Three Cases Treated by, . . 660

Kali Bichromicum. W. P. Wesselhœft, M. D., . . . . . . . . . . . . . 666
Kali bichrom., . . . . . . 67, 103, 285, 413
Kali brom., . . . . . . . . . . . . . . 598
Kali carbonicum, . . . 41, 67, 117, 127, 550, 552, 590
Kali Cyanate. Provers Needed. J. D. Tyrrell, M. D., . . . . . . . . . . . 23
Kali cyanatum, . . . . . . . . . . . . . 18
Kali-iod., . . . . . . . . . . . . . . . 217
Kali muriaticum, . . . . . . . . . . . 172
Kali sulphuricum, . . . . . . . . . . 173
Kalmia, . . . . . . . . . . . . . . . . 206
Kent, J. T., M. D. Abrotanum, with Clinical Cases, . . . . . . . . . . . 58
Kent, J. T. Class-Room Talks, . . . 34
Kent, J. T., M. D. A Criticism of Dr. Holmes, . . . . . . . . . . . . . . . 639
Kent, J. T., M. D. How Symptoms Change, . . . . . . . . . . . . . . 596
Kent, J. T., M. D. The I. H. A. Meeting at Niagara, . . . . . . . . . . . 417
Kent, J. T., M. D. Lecture on Muriatic Acid, . . . . . . . . . . . . . . 12
Kent, J. T., M. D. Reply to Dr. Hughes, 5
Kent, J. T., M. D. Lecture upon Lycopodium, . . . . . . . . . . . . . 462
Kent, J. T., M. D. Lecture upon Podophyllum, . . . . . . . . . . . . 279
Kent, J T., M. D. The Second Prescription, . . . . . . . . . . . . . . 409
Kent, J. T., M. D. Sycosis, . . . . . . 163
Kent, J. T., M. D. Syphilis as a Miasm, . . . . . . . . . . . . . . . 213
Kimball, S. A., M. D. Proceedings of the Organon Society of Boston, . . . 30, 72, 108, 204, 247, 283, 351
Kimball, S. A., M. D. New Members for the I. H. A., . . . . . . . . . . 592
Krause, Wm. H., M. D. A Case of Diphtheria with Convulsions Cured, 271
Kreosotum, . . . . . . . . 67, 143, 273, 573

PAGE

Lachesis, . . . 41, 60, 66, 76, 102, 132, 140, 143, 149, 185, 206, 243, 325, 328, 334, 428, 494, 541, 550, 590, 598, 659
Lac Caninum, An Involuntary Proving of. H. C. Morrow, M. D., . . . . 477
Lac caninum, . . . . 192, 303, 330, 540, 598
Lac vaccinum-defloratum, . . . . . . 106
Laurocerasus, . . . . . . . . . . . 130, 598
Lecture on the *Organon*, . . . . . . . 212
Lectures on Diseases of the Heart. By Alonzo Clark, M. D. Notice of, . . 275
Ledum, . . . . . . . . . . . . . . 143, 269
Lee, Edmund J., M. D. Characteristics as Taught by Hahnemann, . . . . . 451
Lee, E. J., M. D. Clinical Notes on Characteristics, . . . . . . . 80, 144, 218
Lee, E. J., M. D. Clinical Notes on Characteristics. Agaricus Muscar., . 144
Lee, E. J., M. D. Agnus Castus—Clinical Notes on Characteristics, . . . . 150
Lee, E. J., M. D. Ailanthus—Clinical Notes on Characteristics, . . . . . . 218
Lee, E. J., M. D. Aletris Formosa—Clinical Notes on Characteristics, . . 221
Lee, E. J., M. D. Allium Cepa—Clinical Notes on Characteristics, . . . . 223
Lee, E. J., M. D. Allium Sativa—Clinical Notes on Characteristics, . 225
Lee, E. J., M. D. Chronic Diseases, . 335
Lee, E. J., M. D. Editorial Notes, . . 621
Lee, E. J., M. D. Experiments in Therapeutics, . . . . . . . . . . . . 507
Lee, E. J., M. D. The Homœopathic Materia Medica, its Uses and its Imperfections, . . . . . . . . . . 557
Lee, E. J., M. D. Stuttering Speech, . 545
Lee, E. J., M. D. Therapeutics of the Throat. Cistus, . . . . . . . . . . 8
Leggett, S. L. G., M. D. Psora, . . . 159
Light Wanted, . . . . . . . . . . . . 39
Lilienthal, S. Hahnemann's *Organon*. A Query, . . . . . . . . . . . 385
Lilium T., . . . . . . . . . . . 42, 378, 590
Lime Water, . . . . . . . . . . . . . 143
Lithium carb., . . . . . . . . . . 143, 407
Lippe, Adolph, M. D. In Memoriam. Supplement to Feb. No.
Lippe, Adolph, M. D., Some Tributes to the Memory of, . . . . . . . . . 186
Lippe, Adolph, M.D., Memorial Address upon the Life and Work of. P. P. Wells, M. D., . . . . . . . . . . . . 604
Lippe, Library of Dr., for Sale, . . 620, 668
Lobelia, A Note on. C. Carleton Smith, . . . . . . . . . . . . . . . 272
Local Applications. D. C. McLaren, M. D., . . . . . . . . . . . . . . . . 140
Locomotor Ataxy. A Clinical Case. Clarence W. Butler, M. D., . . . . . 651
Lamb's Prize Essays. Review of, . . 333
Long, Samuel, M. D. Homœopathy *vs.* Intermittent Fever, . . . . . . . . 45
Lowe, J. N., M. D. A Clinical Case, with Appendices, . . . . . . . . . . 137
Lutze, F. H., M. D. Answer to Dr. Jamison's Case for Counsel, . . . . 131
Lycopodium, Lecture upon. Prof. J. T. Kent, . . . . . . . . . . . 462, 566
Lycopodium, . . . 18, 125, 137, 154, 181, 238, 274, 305, 312, 362, 366, 413, 462, 467, 499, 505, 551, 554, 570, 575, 580, 590, 619

Magnesia carb., . . . . . . . . . . 42, 67

Magnesia phosphorica, . . . 174, 303, [illegible] 468, 471, 473

Magnesium mur., . . . . . . . . . . . 366

PAGE
Married, . . . . . . . . . . . . . . . . 620
Martin, James T., M. D. Rhus Antidotes, . . . . . . . . . . . . . . . . . 503
Massachusetts Homœopathic Medical Society, Publications of, . . . . . . 386
Masturbation, Excessive Venery, etc. Joseph W. Howe, M. D. Review of, 556
Matteism—The Latest Craze. E. W. Berridge, M. D., . . . . . . . . . . 560
Materia Medica, A. By Samuel Swan, M. D. Review of, . . . . . . . . . 211
McLaren, D. C., M. D. Local Applications, . . . . . . . . . . . . . . . . 140
McLaren, D. C., M. D. A Reply to "Was Hahnemann Inspired?", . . 237
McNeil, A., M. D. A Dietetic Case, . 345
McNeil, A., M. D. A Diagnostic Sign of Cancer, . . . . . . . . . . . . . . 130
McNeil, A., M. D. Lecture on Dulcamara, . . . . . . . . . . . . . . . . 60
McNeil, A., M.D. Mercurial Poisoning, 178
Medical Diagnosis. By J. Graham Brown, M. D. Review of. . . . . . 667
Medical Journals and Practitioners. L. Henderson, M. D., . . . . . . . . . 63
Medical Jurisprudence, A Manual of. By Allen McLane Hamilton, M. D. Review of, . . . . . . . . . . . . . 95
Medorrhinum, . . . . . . . . 167, 185
Mendenhall, Rev. J. K. A Case of Dysentery, . . . . . . . . . . . . . 90
Mental Symptoms, . . . . . . . . . . . 334
Mercurial Poisoning. Dr. A. McNeil, 178
Mercurius, . . . 61, 68, 69, 98, 125, 168, 177, 204, 216, 245, 326, 363, 532, 546, 554
Mezereum, . . . . . . . . . . . . 363, 585
Miasmata, Contagious Diseases Caused by Acute. T. Dwight Stow, . . . . 49
Millefolium, . . . . . . . . . . 143, 209, 407
Miller, J. M., M. D. Some Diagnostic Remarks, . . . . . . . . . . . . . . 615
Missouri Homœopathic Medical College. . . . . . . . . . . . . . . . . 449
Montreal Tracts on Homœopathy. Review of, . . . . . . . . . . . . . . . 668
Morrow, H. C., M. D. An Involuntary Proving of Lac Caninum, . . . . . . 477
Morrow, H. C., M. D. Therapeutic Notes, . . . . . . . . . . . . . . . . 479
Mosch., . . . . . . . . . . . . . . . . . 60
Murex, . . . . . . . . . . . . . . . 23, 280
Muriatic Acid, Lecture on. J. T. Kent, M. D., . . . . . . . . . . . . . . . . 12
Muriatic Acid, . . . . . . . . . . . 282, 571
Myrica, . . . . . . . . . . . . . . . . 67

Naja, . . . . . . . . . . . . . . . . . 445
Nash, Eugene B., M. D. Therapeutics of the Throat, . . . . . . . . . . . . 7
Nash, Eugene B., M. D. Tissue Remedies, . . . . . . . . . . . . . . . . 468
Natrum ars., . . . . . . . . . . . . . . 149
Natrum carb., . . . . . . . . . . . . 363, 590
Natrum mur., . . . 66, 77, 154, 280, 326, 370, 427, 531, 569, 570, 572, 590
Natrum phosphoricum, . . . . . . . . . 174
Natrum sulph., . . . . . . 166, 176, 402, 575
Nebraska State Homœopathic Society, 212
New Jersey State Homœopathic Medical Society. Notice of Meeting of, . 275
New Jersey State Homœopathic Medical Society, The. Review of, . . . 333
New York Homœopathic Medical College, . . . . . . . . . . . . . . . . 334
New York Homœopathic Medical Society, Central, Proceedings of, . . . 100

PAGE
New York Homœopathic Union, Proceedings of the. Clarence Willard Butler, Secretary, . . . . . . . . 299, 486
Niccolum, . . . . . . . . . . . . . . . 69
Nichols, C. F. Populus Candicans, . . 234
Nitric Acid, . . . . . . . . . . 306, 366, 590
Nitrum, . . . . . . . . . . . . . . 40, 445
Norris, Ida F., M. D. Bastinado for Asphyxia, . . . . . . . . . . . . . . 89
Notes and Notices, . . . . 48, 96, 158, 212, 275, 333, 449, 556, 620, 668
Nux Moschata, . . . . . . . . . . 185, 356
Nux Vomica. A. B. Eadie, M. D., . . 423
Nux vomica, . . . . 68, 76, 81, 90, 116, 132, 149, 154, 183, 238, 273, 280, 310, 367, 453, 456, 528, 570, 578, 590, 618, 643, 663

Odium Medicum and Homœopathy. By Dr. John H. Clarke. Notice of, . 332
Ohio Homœopaths, . . . . . . . . . . . 333
Onosmodium virg., . . . . . . . . . . . 595
Ophthalmology. A Journal of, . . . 668
Opium. J. D. Tyrrell, M. D., . . . . . 425
Opium, . . . 118, 130, 149, 209, 316, 367, 439, 546, 570
Opium, The Abuse of, . . . . . . . . 29
*Organon*, Section 153. W. H. A. Fitz, M. D., . . . . . . . . . . . . . . . . 599
*Organon*. Section 153. Selection of Characteristic Symptoms. Edward Cranch, M. D., . . . . . . . . . . . 404
*Organon*, Lecture upon the. Prof. T. F. Allen, M. D., . . . . . . . . . . . 1
Organon Society of Boston. Proceedings of the. S. A. Kimball, M. D., 30, 72, 108, 204, 247, 283, 351
Oranges, Uses of, . . . . . . . . . . . 192

Pathogenetic and Clinical Repertory of the Most Prominent Symptoms of the Head. By C. Neidhard, M. D. Review of, . . . . . . . . . . . . . . 331
Payne, Fred. W., M. D. A Case of Retinitis Pigmentosa, . . . . . . . 286
Peculiar Symptoms, W. S. Gee, M.D., 580
Pellagra and Ustilago Maides, On the Nervous Disturbances of. S. L., . . 430
Peritonitis, Pelvic, Adhesions Following. J. M. Dutton, M. D., . . . . . 383
Petroleum, . . . . . . . . . . . 42, 143, 438
Phosphorus Remedies for Post-Partum Hemorrhage. A. B. Eadie, . . 201
Phosphorus, . . . . 70, 102, 115, 127, 132, 154, 300, 325, 367, 452, 466, 470, 528, 546, 547, 590, 616, 644, 666
Phos. ac., . . . . 127, 153, 155, 281, 572, 590
Physicians' Complete Account Book. G. W. Eschenbach. Notice of, . . . 96
Physiology and Pathology of Diabetes. Notice of, . . . . . . . . . . . . 386
Physostigma, . . . . . . . . . . . 125, 149
Phthisis, The Preferable Climate for, Notice of, . . . . . . . . . . . . . 667
Phytolacca, . . . . . . 66, 67, 143, 196, 590
Picric acid, . . . . . . . . . . . . . . 168
Platina. Post-Partum and other Hemorrhages. A. B. Eadie, M. D., . . . 293
Platina, . . . . . 41, 367, 373, 584, 590, 598
Plumbum, . . . . . . . . . . . . 4, 367, 546
Podophyllum, Lecture upon. J. T. Kent, M. D., . . . . . . . . . . . . 279
Podo., . . . . . . . . . . . . . . . . . 590
Populus Candicans. C. F. Nichols, M. D., . . . . . . . . . . . . . . . . . 234
Post-Graduate Course of Lectures. Notice of, . . . . . . . . . . . . . . 450

PAGE

Potencies, High. Report of a few Cases Terminating Successfully under. J. B. Garrison, M. D., . . . . . . . . . 306
Potencies, High, and Rheumatism. Plea for. Ed. Rushmore, M. D., . . . . . 372
Powel, F., M. D. What is the Remedy? 595
Practical Manual of Gynæcology, A. By G. R. Southwick, M. D. Review of, . . . . . . . . . . . . . . . . . . 157
Prescribing, Art of. Prosper Bender, M. D. Review of, . . . . . . . . . 386
Prescription, the Second. J. T. Kent, M. D., . . . . . . . . . . . . . . . . . 409
Proceedings of the Eighth Annual Session of the International Hahnemannian Association. Notice of, . 448
Proceedings of the Twenty-third Annual Meeting of the Homœopathic Medical Society of Ohio. Review of, . . . . . . . . . . . . . . . . . 211
Proceedings of the State Sanitary Convention of Pennsylvania. Review of, 448
Psora. S. L. G. Leggett, M. D., . . . . 159
Psorinum, . . . . . . . 168, 269, 383, 526
Publications of the Massachusetts Homœopathic Medical Society, 1887. Review of, . . . . . . . . . . . . . 386
Pulsatilla. Remedies for Post-Partum Hemorrhage. Edward Adams, M. D., 202
Pulsatilla, . . . 4, 93, 97, 149, 174, 185, 192, 208, 263, 327, 354, 367, 407, 425, 453, 455, 456, 466, 469, 528, 590, 632
Pulsatilla Nuttalina, . . . . . . . . . 632

Queries as to Rhus and other Poisonings. Fred. Hooker, M. D., . . . 384
Quinine Curse, The. Wm. Jefferson Guernsey, M. D., . . . . . . . . . . 72
Quinine, . . . . . . . . . . . . . . . . 205

Rannuculus bulbosus, . . . . . . . . 407
Raph., . . . . . . . . . . . . . . . . . 575
Ratan., . . . . . . . . . . . . . . . . 185
Rejoinder to Dr. Hughes, by Prosper Bender, M. D. Review of, . . . . . 668
Remittent Fever, A Case of. J. C. Holloway, M. D., . . . . . . . . . . . . 274
Removals, . . . . 48, 275, 333, 449, 556, 668
Repertory of Characteristics, The, . . 57
Repertory of Gonorrhœa. By Samuel A. Kimball, M. D. Review of, . . . 332
Repertory of Head Symptoms, Pathogenetic and Clinical. C. Neidhard, M. D., . . . . . . . . . . . . . . . . 331
Reply to Dr. Hughes. J. T. Kent, M. D., . . . . . . . . . . . . . . . . . 5
Report of a Few Cases Terminating Successfully Under the Administration of the Higher Potencies. J. B. Garrison, M. D., . . . . . . . . . . . 306
Respiratory Symptoms, Some, . . . . 504
Retinitis Pigmentosa. A Case of. Frederick W. Payne, M. D., . . . . . . . 286
Rheumatism, and a Plea for the Higher Potencies. Edw. Rushmore, M. D., 372
Rhododendron, . . . . . . . . . 60, 553
Rhus and other Poisonings, Queries as to. Fred. Hooker, M. D., . . . . . . 384
Rhus Antidotes. James T. Martin, M. D., . . . . . . . . . . . . . . . . 503
Rhus Rad. *vs.* Rhus Tox. Robert Farley, M. D., . . . . . . . . . . . . . 313
Rhus rad., . . . . . . . . . . . . . . 266
Rhus tox., . . . 60, 62, 69, 132, 143, 169, 196, 209, 265, 266, 354, 455, 465, 554, 590, 618, 662

PAGE

Rochester Hahnemannian Society, The. Proceedings of. W. H. Baker, M. D., . . . . . . . . . 242, 360, 496, 538
Rumex crispus, . . . . . . . . . . . 42, 554
Rushmore, Edward, M. D. Rheumatism, and a Plea for the Higher Potencies, . . . . . . . . . . . . . . . 372
Ruta grav., . . . . . . . . 61, 149, 185, 367

Sabad, . . . . . . . . . . . . . . . . . 590
Sabina. Remedies for Post-Partum Hemorrhage. W. J. Hunter Emory, M. D., . . . . . . . . . . . . . . . . 204
Sabina, . . . . . . . . . . . . . . . . . 445
Salicylic acid, . . . . . . . . . . . . 207
Salient Materia Medica and Therapeutics. By C. L. Cleveland, M. D. Notice of, . . . . . . . . . . . . . . . 275
Sanguinaria, . . . . . . . . . . . . . 633
Sanicula, . . . . . . . . . . . . . . . 185
Sapo, . . . . . . . . . . . . . . . . . 143
Sarsap., . . . . . . . . . . . . . . . . 571
Sartor Resartus. M. W. Vandenburg, A. M., M. D., . . . . . . . . . . . . 278
Schmitt, Julius G., M. D. Proceedings of the Central New York Homœopathic Medical Society, . . . . . . 100
Scientific Medication and Specific Medicines. By Dr. John M. Scudder. Review of, . . . . . . . . . . . . 211
Secale. Remedies for Post-Partum Hemorrhage. J. D. Tyrrell, M. D., . 199
Secale, . . . . . . . . . . . . . . . . 546
Second Prescription, The. J. T. Kent, M. D., . . . . . . . . . . . . . . . 409
Selection of Characteristic Symptoms, Hahnemann's *Organon*, Paragraph 153. Edward Cranch, M. D., . . . . 404
Selenium, . . . . . . . . . . . 155, 328, 546
Senega, . . . . . . . . . . . . . . . . 67
Sepia. Therapeutics of the Throat. B. L. B. Baylies, M. D., . . . . . . 231
Sepia, . . . 149, 156, 263, 274, 280, 281, 302, 355, 367, 452, 464, 528, 549, 569, 590
Sherbino, G. W., M. D. Some Clinical Notes on Aqua Sanicula, . . . . . . 439
Sherbino, G. W., M. D. Therapeutics of the Throat. Baptisia Tinctoria, . 135
Sherbino, G. W., M. D. Some Clinical Cases, . . . . . . . . . . . . . . . . 179
Sherbino, G. W., M. D. Texas Homœopathic Medical Society, . . . . . . 304
Silicea, . . . 98, 141, 149, 155, 172, 173, 239, 309, 324, 368, 569, 590, 615, 660
Simmons, B., M. D. Cases from Practice, . . . . . . . . . . . . . . . . 647
Skin Diseases, Fallacious and True Theories Concerning Certain. Dr. H. Goullon, . . . . . . . . . . . . . 480
Skin Diseases, Fox's Atlas of. Notice of, . . . . . . . . . . . . . 157, 211, 333
Similia Similibus Curantur. By Chas. T. Mack, M. D. Review of, . . . . . 157
S. L. A Case of Fatal Poisoning by Chininum Sulph., . . . . . . . . . . 179
S. L. A Correction, . . . . . . . . . . 384
S. L. Notes on a Dietetic Case, . . . . 479
S. L. Stuttering Speech, . . . . . . . 597
S. L. A Case of Chronic Sulphur Poisoning, . . . . . . . . . . . . . . . . 314
S. L. A Centre-Shot with Sulphur, . . 664
S. L. G. L. Class-Room Talks, from Lectures by Professor Kent, . . . 34, 97, 239, 324, 343, 474
S. L. G. L. Lecture upon Lycopodium, from Lecture of Professor Kent, 462, 566

PAGE

Smith, C. Carleton, M. D. Clinical Notes on Characteristics, . 80, 144, 150, 218, 221, 223, 225, 272
Smith, C. Carleton, M. D. Chelidonium, . . . . . . . . . . . . . . . . . 185
Spalding, C. W., M. D. Forces Engaged in the Circulation of the Blood, . . 542
Spalding, C. W., M. D. Glands, . . . 458
Spigelia, . . . . . . . . . . . 185, 206, 599, 616
Spongia, . . . . . . . . . . . . . . 124, 156
Stannum, . . . . . . . . . . . . . . 132, 590
Staphisagria, . . . 98, 130, 132, 144, 155, 590
State Sanitary Convention of Penna., Proceedings of. Review of, . . . . . 448
Sticta, . . . . . . . . . . . . . . . . 150
Still, Horace, M. D. Ambra Grisea, . 126
Stillingia, . . . . . . . . . . . . . . . 492
Stoaks, F. E., M. D. Clinical Cases, . . 438
Stow, T. Dwight, M. D. Contagious Diseases, Caused by Acute Miasmata, 49
Stramonium. J. D. Tyrrell, M. D., . . 260
Stramonium. Post-Partum and other Hemorrhages. A. B. Eadie, M. D., . 294
Stramonium, . . . 132, 150, 170, 210, 328, 539, 546, 571, 584
Stuttering Speech. E. J. L., . . . . . 545
Stuttering Speech. S. L., . . . . . . . 596
Sulphur, A Centre Shot with. S. L. . 664
Sulphur, . . . 4, 19, 60, 72, 102, 140, 155, 168, 182, 184, 246, 271, 290, 324, 353, 359, 363, 368, 370, 373, 379, 519, 553, 573, 574, 580, 590, 644
Sulphur Poisoning, A Case of Chronic. S. L., . . . . . . . . . . . . . . 314
Sulphur in Melancholia. J. M. Dutton, M. D., . . . . . . . . . . . 380
Sulphuric Acid. Post-Partum and other Hemorrhages. A.B.Eadie,M.D., 294
Sulph. acid, . . . . . . . . . . . . . . 368
Sumbul, . . . . . . . . . . . . . . . . 67
Surgery, Too much, . . . . . . . . 62, 276
Surgical Journal, A, . . . . . . . . . 212
Sycotic Warts, . . . . . . . . . . . . 435
Sycosis, Some Symptoms of. Geo. H. Clark, M. D., . . . . . . . . . . . 398
Sycosis J. T. Kent, M. D., . . . . . 163
Symphytum, . . . . . . . . . . . . 143, 172
Symptoms Change, How. J. T. Kent, M. D., . . . . . . . . . . . . . . . . 596
Symptoms not Diseases, . . . . . . . . 334
Syphilinum, . . . . . . . . . . . . 168, 319
Syphilis as a Miasm. J. T. Kent, M. D., 213
Syracuse Hahnemannian Club. Frederick Hooker, M. D., . . 197, 262, 357, 491, 641
Tabacum, . . . . . . . . . . . . . . 525, 546
Tabes Dorsalis and Aluminum Metallicum. Bœnninghausen, . . . . . . 526
Taking the Case. Robt. Farley, M. D., 25
Taraxacum, . . . . . . . . . . . . . . 303
Tarent, . . . . . . . . . 150, 326, 573, 584
Tellurium, . . . . . . . . . . . . . . . 185
Terebinthina, . . . . . . . . . . . . 144, 573
Testimony for High Potencies, . . . . 334
Texas Homœopathic Medical Society. G. W. Sherbino, M. D., Secretary, . . 304
Thayer, W. J., D. D. S., M. D. Dietetics, Its Bearing on Special and General Tissue Building, . . . . . . . . . . 346
Therapeutics of the Throat. E. B. Nash, M. D., . . . . . . . . . . . . 7
Therapeutics of the Throat. Æsculus Hipp. John V. Allen, M. D., . . . . 64
Arum Triphyllum. Wm. Jefferson Guernsey, M. D., . . . . . . . . 132
Ailanthus, John V. Allen, M. D., . 67
Therapeutics of the Throat. Baptisia Tinctoria. Geo. W. Sherbino, M. D., 135
Therapeutics of the Throat. Carbo Animalis. E. Cranch, M. D., . . . 226
Therapeutics of the Throat. Carbo veg. E. Cranch, M. D., . . . . . . 228
Therapeutics of the Throat. Cistus E. J. Lee, M. D., . . . . . . . . . . . . . 8
Therapeutics of the Throat. Ignatia. Geo. H. Clark, M. D., . . . . . . . . 9
Therapeutics of the Throat. Rhus Tox. C. E. Chase, M. D., . . . . . . . . . 193
Therapeutics of the Throat. Sepia. B. L. B. Baylies, M. D., . . . . . . . . 231
Therapeutic Notes. H. C. Morrow, M. D., . . . . . . . . . . . . . . . . 479
Therapeutics, Experiments in. E. J. Lee, M. D., . . . . . . . . . . . . . 507
Thea, . . . . . . . . . . . . . . . . . . 150
Theism, A New Disease, . . . . . . . 38
Theridion, A Note on. Dr. Baruch, . 331
Theridion, . . . . . . . . . . . 132, 371, 649
Throat, Therapeutics of. See "Therapeutics of the Throat."
Thuja, . . . 166, 177, 354, 368, 403, 454, 523, 525, 532, 552
Tissue Remedies. Eugene B. Nash, M. D., . . . . . . . . . . . . . . . . 468
Tissue Remedies. Review of, . . . . 158
Tongue, Indented, . . . . . . . . . . . 446
Tracheotomy. A Plea for Natural Death. Stuart Close, M. D., . . . . 340
Tracts on Homœopathy, Montreal, Review of, . . . . . . . . . . . . . . . 668
Tracts, Homœopathic League. Notice of, . . . . . . . . . . . . . . . 96, 332
Transactions of the American Institute of Homœopathy. Dr. J. C. Burgher. Review of, . . . . . . 48, 667
Transactions of the Homœopathic Medical Society of Penna. Review of, 96
Treatment, Complete Hand-Book of. By Wm. Aitkin, M. D. Notice of, . 48
Tributes to the Memory of the Late Dr. Lippe, . . . . . . . . . . . . . . . 187
Twelve Tissue Remedies of Schussler, The. By Drs. Wm. Bœricke and W. A. Dewey. Review of, . . . . . . 158
Typhoid Fever, George H. Clark, M. D., . . . . . . . . . . . . . . . . . 635
Tyrrell, J. D., M. D. Apis Mellifica. Post-Partum and other Hemorrhages, 296
Tyrrell, J. D., M. D. Calcarea. Remedies for Post-Partum Hemorrhage, . 120
Tyrrell, J. D., M. D. Chamomilla. Post-Partum and other Hemorrhages, . . 295
Tyrrell, J. D., M. D. The Hahnemann Club of Toronto, . . . . . . . . 74, 251, 291
Tyrrell, J. D., M. D. Kali Cyanate. Provers Needed, . . . . . . . . . . 23
Tyrrell, J. D., M. D. Opium, . . . . 225
Tyrrell, J. D., M. D. Proceedings of the Hahnemann Club of Toronto, 116, 198, 420
Tyrrell, J. D., M. D. Secale. Remedies for Post-Partum Hemorrhage. . . . 199
Tyrrell, J. D., M. D. Stramonium, . . 260

Urine, Zinc in Hysterical Retention of, 650
Urtica urens, . . . . . . . . . . . . . . 144
Ustilago. Remedies for Post-Partum Hemorrhage. L. Hamilton Evans, M. D., . . . . . . . . . . . . . . . . 200
Ustilago, Maides and Pellagra. On the Nervous Disturbances of. S. L., . . 430
Ustilago, . . . . . . . . . . . . . . . . 155

PAGE

Vaccination, Death after. William Young, . . . . . . . . . . . . . . . . . 544
Vandenburg, N. W., M. D. Was Hahnemann Inspired? . . . . . . . . . 77
Vandenburg, N. W., M. D. A Partial Repertory of Hypochondriasis, . . . 589
Vandenburg, N. W., A. M., M. D. Sartor Resartus, . . . . . . . . . . . . 278
Vandenburg, N. W., M. D. How to Find the Drug, . . . . . . . . . . . . 632
Veratrum in Cholera Morbus. H. P. Holmes, M. D., . . . . . . . . . . 602
Veratum alb., . . . 209, 368, 496, 553, 570, 632
Verat viride, . . . . . . . . . . . . 584, 632
Verifications, a few. H. G. Glover, M. D., . . . . . . . . . . . . . . . . . 618
Verifications of Symptoms. Flora A. Waddell, M. D., . . . . . . . . . . . 377
Vinca minor, . . . . . . . . . . . . . . . 127
Vipera, . . . . . . . . . . . . . . . . . 546
Visiting List. Otis Clapp & Son's, . . 668
Visiting List. Dr. Faulkner's, . . 158, 668
Vital Force, The. W. A. Hawley, M. D., . . . . . . . . . . . . . . . . . . . 368

Waddell, Flora A., M. D. Verifications of Symptoms, . . . . . . . . . . . . . 377
Was Hahnemann Inspired? N. W. Vandenburg, A. M., M. D., . . . . . 77
Was Hahnemann Inspired? A reply to. D. C. McLaren, M. D., . . . . . . . 237

PAGE

Wells, P. P., M. D. The Dose, . . . . 623
Wells, P. P., M. D. The Examination of the Patient for a Homœopathic Prescription, . . . . . . . . . . . . . 509
Wells, P. P., M. D., Memorial Address upon the Life and Work of Adolph Lippe, . . . . . . . . . . . . . . . 605
Wells, P. P., M. D. What is a Proving? . . . . . . . . . . . . . . . . 277
Wesselhœft, Wm. P., M. D. Address before the International Hahnemannian Association at its Ninth Annual Meeting, . . . . . . . . . . . . . 387
Wesselhœft, W. P., M. D. Aloe Socotrina, an Anti-psoric Remedy, . . . 576
Wesselhœft, W. P., M. D. Kali Bichromicum, . . . . . . . . . . . . . 666
Wesselhœft, Wm. P., M. D. Translation of Bœnninghausen's *Aphorisms*, 32
What!! . . . . . . . . . . . . . . . . 212
What is a Proving? P. P. Wells, M. D., . . . . . . . . . . . . . . . . . . . 277
What is the Remedy? . . . . . . . . 543
What is the Remedy? F. Powel, M. D., 595
Wilmington Homœopathic Hospital. Notice of, . . . . . . . . . . . . . . . 276

Young, William. Death after Vaccination, . . . . . . . . . . . . . . . . . 544

Zincum, . . . . . . 155, 527, 590, 599, 650

VOL. VIII. JANUARY, 1888. No. 1.

### THE HOMŒOPATHIC PHYSICIAN,

A Monthly Journal of Homœopathic Materia Medica and Clinical Medicine.

"*He is a freeman whom truth makes free, and all are slaves beside.*"
"*Seek the Truth: come whence it may, cost what it will.*"

235.

EDITED AND PUBLISHED BY

EDMUND J. LEE, M. D.,

AND

WALTER M. JAMES, M. D.

NOTICE.

***All articles for publication, books for review, business communications, etc., must be sent to Editors, 1123 Spruce Street, Philadelphia.***

***Subscribers will please notice that this Journal will be sent until arrears are paid and it is ordered discontinued.***

PHILADELPHIA:

No. 1123 SPRUCE STREET.

LONDON:
ALFRED HEATH & CO., SOLE AGENTS FOR GREAT BRITAIN,
114 Ebury Street, Eaton Square.

☞ ***Yearly Subscription, $2.50, invariably in advance. Single numbers, 25 cts.***

Entered at the Philadelphia Post-Office as Second-Class Mail Matter.

***Subscribers are respectfully requested to make all checks and money-orders payable to THE HOMŒOPATHIC PHYSICIAN, and not to either of the Editors.***

### CONTENTS.

***MISCELLANEOUS.*** PAGE

Lecture upon the Organon. Professor T. F. Allen, . . . . . 1

Fresh Air: Dyspnœa, . . . . . . . . . . . . . 4

Reply to Dr. Hughes. Professor J. T. Kent, . . . . . . 5

The Hahnemann Club of Toronto, . . . . . . . . . 6

Therapeutics of the Throat. E. B. Nash, M. D., . . . . . 7

Therapeutics of the Throat: Cistus. E J. Lee, M. D., . . . . 8

Therapeutics of the Throat: Ignatia. Geo. H. Clark, M. D., . . 9

Lecture on Muriatic Acid. Professor J. T. Kent, . . . . . 12

Proceedings of the Hahnemannian Association of Pennsylvania, . 17

Asterias Rubens. Geo. H. Clark, M D., . . . . . . . 20

Kali Cyanate: Provers Needed. J. D. Tyrrell, M. D., . . . . 23

Taking the Case. Robert Farley, M. D., . . . . . . . . 25

The Abuse of Opium, . . . . . . . . . . . . . 29

Proceedings of the Organon Society of Boston, . . . . . . 30

Mental Symptoms Important, . . . . . . . . . . 32

Bœnninghausen's Aphorisms, . . . . . . . . . . 32

Class-Room Talks, No. 2. Professor Kent, . . . . . . . 34

The Hospitals of the Women's Homœopathic Association of Penna., 37

Light Wanted, . . . . . . . . . . . . . . . 38

Theism a New Disease, . . . . . . . . . . . . 39

Clinical Notes on Characteristics, . . . . . . . . . 39

Clinical Confirmation of High Potencies. B. L. B. Baylies, M. D., . 40

Prolonged Constipation. W. S. Gee, M. D., . . . . . . 43

Homœopathy *vs.* Intermittent Fever. Samuel Long, M. D., . . . 45

***BOOK NOTICES AND REVIEWS.***

